# Enhancing Perception Through Context-Adaptive Visible and SWIR Image Fusion in Harsh Environments

**DOI:** 10.3390/s26134035

**Published:** 2026-06-25

**Authors:** Alexandre Riffard, Mathieu Labussière, Pierre Duthon, Romuald Aufrère

**Affiliations:** 1 Université Clermont Auvergne, Clermont Auvergne INP, CNRS, Institut Pascal, F-63000 Clermont-Ferrand, France; mathieu.labussiere@uca.fr (M.L.); romuald.aufrere@uca.fr (R.A.); 2 Cerema, Research Team “Intelligent Transport Systems”, F-63017 Clermont-Ferrand, France; pierre.duthon@cerema.fr

**Keywords:** autonomousvehicles, short-wave infrared (SWIR), image fusion, adverse weather, no-reference image quality assessment (NR-IQA)

## Abstract

Robust perception in adverse weather conditions remains a significant challenge for autonomous vehicles. Short-wave infrared (SWIR) sensors offer specific physical properties that enable them to penetrate atmospheric disturbances like fog, rain, and snow. However, effectively combining this robustness with the textural and colour information of visible (VIS) cameras is difficult due to signal decorrelation and the limitations of static fusion schemes. To address this, we present VISWIR (Visible and SWIR Weighted Image Reconstruction), a pixel-level fusion method based on a multi-scale pyramid architecture. We introduce an automated strategy for scheduling parameters based on weather conditions using an optimisation framework. Rather than relying on static weights, our method applies offline parameter scheduling to adjust fusion hyperparameters based on the meteorological context. We focus on a multi-objective optimisation approach that maximises perceptual image quality via No-Reference Image Quality Assessment (NR-IQA) metrics. Validated in controlled environment scenarios with varying weather severities, our results confirm the potential of VISWIR as a robust, lightweight algorithmic baseline to enhance the perception capabilities of autonomous vehicles in adverse weather conditions.

## 1. Introduction

The ability of autonomous vehicles (AVs) to operate safely in adverse weather is a critical factor in extending their Operational Design Domain (ODD). Current perception systems, relying mainly on visible spectrum (VIS) sensors, can suffer severe performance degradations in fog, rain, smoke, or glare, leading to reduced detection range and accuracy [1,2,3]. This limitation poses a significant challenge for robust perception and safe navigation in unstructured or harsh environments.

**Figure 1:**
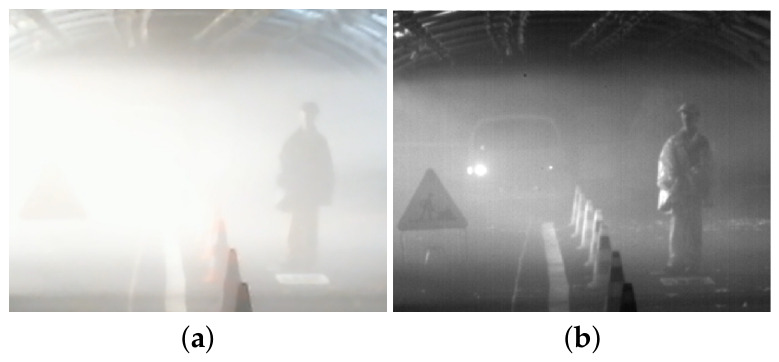
Artificial fog conditions during daylight at the Cerema’s PAVIN platform [8]. The same scene is captured (**a**) in the visible spectrum (0.38–0.75 µm), and (**b**) in the Short-Wave Infrared (SWIR) spectrum (0.9–1.7 µm). Reproduced from [9].

Short-Wave Infrared (SWIR) imaging, typically defined within the 0.9 to 1.7 µm wavelength range but broadly extending from 0.7 to 2.5 µm, offers specific properties for AV perception [4]. Its atmospheric penetration capabilities enable the capture of scene details that are invisible in the visible spectrum, particularly under degraded visibility conditions. As illustrated in Figure 1, SWIR can reveal critical features obscured in visible images, making it a promising modality for all-weather perception. SWIR imaging has an established track record in military and surveillance applications [5,6,7], where robust perception under adverse conditions is critical. These successes demonstrate its potential for safety-critical domains such as autonomous driving.

**Figure 2:**
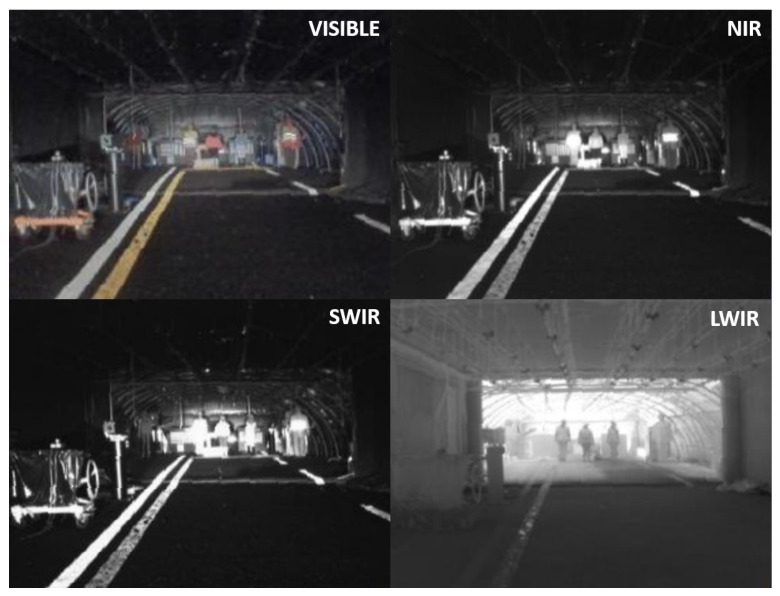
Visual comparison of the same scene captured in four spectral bands: Visible (VIS), Near-Infrared (NIR), Short-Wave Infrared (SWIR), and Long-Wave Infrared (LWIR). The figure illustrates the differences in appearance, contrast, and detail visibility across these modalities. Images acquired by Cerema as part of the FUI AWARE project. Adapted from [11].

Historically, adoption in civil applications was limited by the high cost of sensors, favouring other spectra among the infrared band, such as Long-Wave Infrared (LWIR; 8–12 µm) and Near-Infrared (NIR; 0.75–1 µm) (see Figure 2). However, recent advances, notably quantum dot-based detectors leveraging CMOS technology, have reduced costs while improving resolution and sensitivity [10], paving the way for integration into automotive sensing suites.

Fusing VIS and SWIR images combines the SWIR’s robustness in adverse conditions with the visible spectrum’s colour information, which is crucial for object recognition and scene interpretation, particularly in poor visibility [12,13]. However, publicly available SWIR datasets remain scarce [14] and few fusion algorithms are tailored to its spectral characteristics, especially in the context of mobile robotics and AV applications [15]. In this study, we focus on pixel-level fusion (i.e., early fusion), which enables data to be integrated at an early stage to maximise the spectral information.

### 1.1. Related Work

Currently, most infrared fusion methods focus on the LWIR or NIR spectra, which often fail to fully exploit SWIR’s potential in harsh weather.

#### 1.1.1. VIS–LWIR Fusion

Fusion methods using LWIR are well established and widely used, particularly in surveillance and object detection applications [16]. LWIR captures thermal emissions, but more importantly, its long wavelength significantly exceeds the typical size of fog and smoke particles. This physical characteristic makes it highly robust to atmospheric scattering, enabling clear perception in rain and fog.

**Multi-scale fusions** [13] (e.g., Wavelet Transform-Based Fusion [17]) are frequently used to combine fine visible textures with the thermal contrasts provided by LWIR. This enables efficient detail capture and preservation of the overall structure while highlighting thermal information. However, conventional multi-scale schemes based on fixed transforms can be less effective in complex environments dominated by non-linear structures, where their ability to adapt to local variations is limited.
**Neural networks** are particularly popular in surveillance systems, where high quality is crucial (e.g., modern night-vision systems). However, they require large datasets to guarantee high-quality training. GAN-based approaches such as TarDAL [18], have shown improved detail preservation and robustness in VIS–LWIR fusion for adverse conditions, but their design is tailored to LWIR characteristics and would require adaptation for SWIR imagery. More recently, deep learning-based LWIR fusion methods have been proposed for enhanced perception in complex outdoor scenes [19], further illustrating the maturity of VIS–LWIR fusion compared to VIS–SWIR.
**Saliency-based methods** [20] highlight visually prominent regions to create saliency maps, preserving the integrity of salient objects and enhancing the visual quality. These methods, which often combine saliency analysis and filtering, effectively retain important information but can be complex to implement and require optimisation for different scenarios.

Even when optimised for LWIR, such methods are not directly applicable to SWIR due to fundamental differences in spectral characteristics. Most importantly, LWIR is an emissive modality (capturing thermal radiation), whereas SWIR is a reflective modality (capturing reflected photons, similar to the visible spectrum). Consequently, LWIR images lack natural shadows and reflective textures, such as painted road markings or traffic signs, and the appearance of objects varies with ambient thermal conditions rather than their physical surface properties. In contrast, SWIR preserves these crucial reflective details, making it significantly more intuitive for standard scene understanding. Moreover, LWIR cameras are typically of lower resolution, bulkier, and historically more costly.

#### 1.1.2. VIS–NIR Fusion

The NIR spectrum has interesting properties in terms of atmospheric penetration, such as resistance to haze and smoke. VIS–NIR fusion methods have shown promising potential for applications requiring enhanced perception in low light or haze conditions [21].

**Complementary information-based fusion** [22,23] analyses the differences between visible and NIR spectra at the physical signal level to design a complementary fusion model. This approach leverages the unique characteristics of each spectrum to enhance the overall image quality by integrating complementary information. This method aims to create a more comprehensive representation, improving the robustness and detail retention in the fused images. Morphological approaches, such as Top-Hat [23], enhance local contrast and fine structures while preserving colours, with structuring element size automatically guided by granulometric analysis.
**Pyramid transform** [24] is a specific multi-scale approach designed to provide smooth transitions between spectra. It offers effective preservation of spectral detail and produces natural-looking images suitable for visualisation. Compared to traditional multi-scale methods, it can better handle gradual variations, but still requires careful parameter tuning to avoid inconsistent fusion and the appearance of artefacts.
**Neural networks** [25] are particularly effective for complex environments. However, similarly to LWIR, they require a large volume of training data, which is not always available.

Despite these advantages, the NIR spectrum still shows limitations in harsh conditions, where SWIR can provide unique benefits, for example, in the presence of smoke.

#### 1.1.3. VIS–SWIR Fusion

SWIR-specific fusion solutions remain scarce, partly due to the lack of publicly available datasets for this spectrum [7].

Existing methods using NIR or LWIR are often designed to maximise spatial or thermal details. As a result, they require significant adjustments or entirely new strategies to handle the specific characteristics of SWIR imagery. Among the few dedicated solutions, V-SWIR-IF [26] combines intensity-based registration using Mattes Mutual Information (MMI) with a 1 + 1 evolutionary optimiser and Discrete Wavelet Transform (DWT) fusion, followed by RGB reconstruction, to produce visually natural results. While effective on the authors’ own dataset, its design, relying on per-channel DWT fusion and a registration stage optimised for static scenes, is less suited to our target context of embedded perception in adverse weather. Another example is dual-band polarisation fusion [27], proposed for specific imaging contexts using a VIS–SWIR polarisation sensor.

A more recent VIS–SWIR study [28] reports competitive no-reference quality metrics compared to visible–thermal baselines, but the absence of dataset, code, and camera specifications limits the reproducibility and generalisability of its results. To the best of our knowledge, no existing method addresses full-spectrum VIS-SWIR fusion, especially for mobile robotics. Most target a narrow SWIR sub-band [29] or static surveillance scenarios [7], restricting their applicability to embedded navigation and perception.

Furthermore, while modern deep learning architectures (e.g., Convolutional Neural Networks and Generative Adversarial Networks) dominate other multimodal fusion tasks, their application to VIS-SWIR fusion is severely hindered by the data bottleneck [2,14]. Purely data-driven models require massive volumes of paired, pixel-aligned images captured under a wide diversity of adverse weather conditions, a resource that is currently non-existent in the public domain. Beyond data scarcity, heavy neural networks typically operate as opaque black boxes, lacking the physical interpretability and mathematical guarantees necessary for safety-critical autonomous driving [30,31]. Consequently, they are notoriously susceptible to catastrophic performance drops under out-of-distribution domain shifts, such as encountering weather severities not explicitly represented in their training set [32]. These severe extrapolation shortcomings highlight the need for physically grounded approaches in safety-critical applications. Unlike unconstrained learning models, frameworks leveraging known optical priors and mathematical bounds can maintain structural stability and predictable behaviour regardless of environmental severity. Finally, it is important to emphasise that among the few existing VIS-SWIR fusion methods, none currently provide open-source implementations, complicating fair comparisons and the establishment of robust baselines.

### 1.2. Aim and Contributions

To address the limitations of current perception systems under adverse weather, this study aims to develop a robust image fusion pipeline leveraging the complementary properties of the visible and SWIR spectra. Specifically, we propose **VISWIR** (Visible and SWIR Weighted Image Reconstruction), a pixel-level fusion framework tailored for outdoor robotic perception.

Rather than proposing a fundamentally new architectural paradigm, the main contributions of this work lie in the physical adaptation of existing multi-scale concepts to the specific constraints of the SWIR domain, and are summarised as follows:

- **A physical adaptation for VIS-SWIR fusion:** We adapt a weight-map-guided multi-scale pyramid architecture to handle the highly decorrelated nature of SWIR data. While building upon foundational concepts originally designed for the NIR band [24], the essential difference lies in our specific physical adaptations for the SWIR spectrum (e.g., tailored pre-processing, weather-driven weighting, and specific tone mapping). This translates the framework to process SWIR signals effectively, revealing structural details completely obscured to RGB sensors in degraded conditions.
- **Weather-driven parameter scheduling:** We address the limitations of fixed-parameter fusion by formulating the hyperparameter selection as a multi-objective optimisation problem. Using an automated offline strategy guided by non-reference perceptual metrics (NR-IQA), we derive context-specific parameter sets. This allows the fusion framework to schedule its parameters based on the meteorological context, shifting away from empirical static weights.
- **Empirical validation as a lightweight baseline:** We demonstrate the effectiveness and algorithmic efficiency of the proposed method through evaluations in controlled weather facilities (PAVIN). Furthermore, preliminary assessments on a dynamic, real-world driving dataset (RASMD) indicate that VISWIR can consistently recover local contrast and suppress sensor noise while maintaining natural colour fidelity, establishing it as a robust algorithmic baseline for embedded perception.

Our source code will be made publicly available at: https://github.com/comsee-research/VISWIR (accessed on 22 June 2026). The datasets generated and/or analysed during the current study are available from the corresponding author upon reasonable request.

### 1.3. Organisation of the Paper

The remainder of this paper is organised as follows. First, Section 2 details the proposed VISWIR framework, including the multi-scale pyramid fusion architecture and the weather-driven parameter optimisation strategy. Following up, Section 3 describes the experimental setup, the acquired multi-modal dataset, the comparative baselines, and the evaluation metrics. Then, Section 4 presents the qualitative and quantitative evaluations of the proposed method against state-of-the-art approaches. The results are discussed in Section 5, along with an assessment of dynamic scenarios. Finally, Section 6 concludes the paper and outlines future research directions.

## 2. Methodology

This section presents the core components of the proposed approach. First, it details the VISWIR framework, a multi-scale image fusion pipeline designed to effectively combine visible and SWIR data. Second, it describes the automated, weather-driven optimisation strategy employed to configure the fusion hyperparameters offline according to the environmental context. Note that while this section focuses on the architectural and mathematical formulation of the pipeline, an analysis of its computational complexity and exact frame processing times (e.g., 0.70 s per frame on a standard CPU) is provided later in Section 5.

### 2.1. Proposed VISWIR Framework

**Figure 3:**
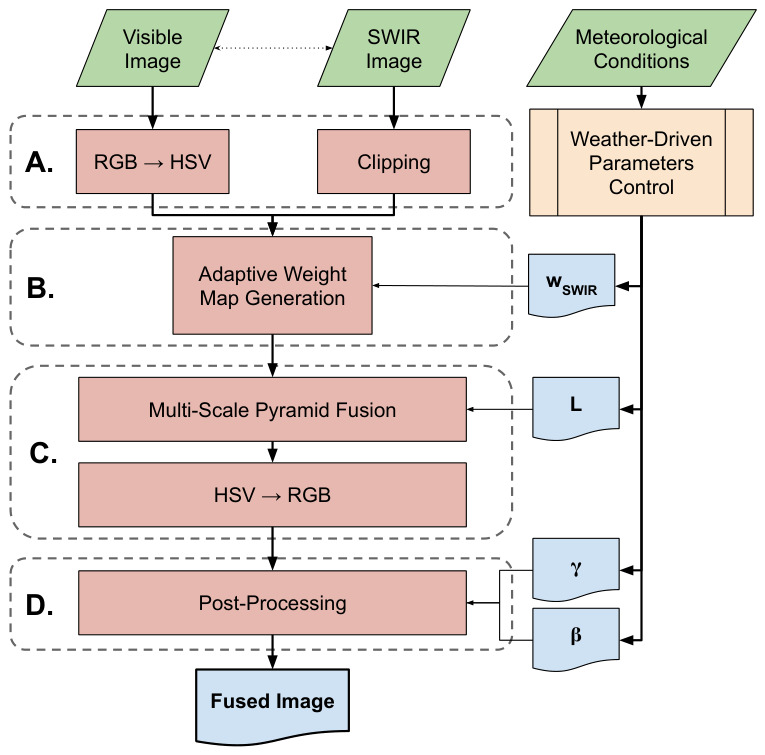
Processing pipeline of our VISWIR fusion method. Unlike static approaches, the process is governed by a context-aware LUT scheduling based on weather-dependent parameters ($\Theta =\{w_{\mathrm{SWIR}},L,\beta ,\gamma \}$). (**A.**) Image pre-processing; (**B.**) Adaptive weight map calculation guided by $w_{\mathrm{SWIR}}$; (**C.**) Multi-scale pyramid fusion defined by depth *L*; and (**D.**) Post-processing adjusted by tone-mapping factors β and γ to match environmental conditions.

The proposed VISWIR framework performs an adaptive fusion of visible and SWIR images. A key feature of this approach is the direct integration of meteorological conditions into the fusion hyperparameters. Rather than relying on static parameters, the algorithm operates as a context-adaptive fusion framework. As illustrated in Figure 3, the pipeline uses a set of external control parameters, $\Theta =\{w_{\mathrm{SWIR}},L,\beta ,\gamma \}$, which are adjusted according to the current weather measurement. This method builds upon the “Pyramid Transform” framework [24], which we have significantly modified to accept these context-aware control parameters and to handle the specific decorrelation of SWIR data.

The fusion method consists of four main phases:

- Images pre-processing.
- Adaptive weight maps calculation and normalisation.
- Multi-scale pyramid fusion.
- Weather-aware post-processing.

Concretely, for a pair of aligned visible and SWIR images, we recreate a pseudo-image of the luminance based on a weighted combination of the visible luminance (Value) and the SWIR image in a pyramid framework to capture details at different scales. The goal is to replace the original luminance of the visible image with the computed luminance of the pseudo-image.

#### 2.1.1. Images Pre-Processing

Our method takes as input a pair of images previously aligned and visually registered.

**SWIR pre-processing.** Unlike standard VIS-NIR fusion, where raw contrast is often sufficient, SWIR imagery in adverse weather suffers from severe histogram compression and sensor-specific noise. Data preparation is therefore a necessary step. Initial processing is carried out directly via the camera parameters by selecting an adapted NUC (Non-Uniformity Correction). We then explicitly enhance the image contrast and dynamic range by applying a clipping and histogram stretching procedure. Specifically, to eliminate extreme outliers (e.g., sensor hot pixels) without compromising the core signal, we compute a dynamic clipping threshold τ=μ+2.576σ, where μ and σ are the global mean and standard deviation of the raw SWIR image. This threshold statistically bounds 99% of the normal intensity distribution. The raw image *I* is first clipped such that $I_{\mathrm{clipped}}\left(x,y\right)=\min\left(I\left(x,y\right),\tau \right)$. Following this, a standard Min–Max normalisation redistributes the clipped intensities across the full 8-bit dynamic scale:
  (1) $$I_{\mathrm{SWIR}}\left(x,y\right)=255\times \frac{I_{\mathrm{clipped}}\left(x,y\right)-\min\left(I_{\mathrm{clipped}}\right)}{\max\left(I_{\mathrm{clipped}}\right)-\min\left(I_{\mathrm{clipped}}\right)}$$
  This operation removes extreme pixel values and redistributes the remaining intensity range over the full dynamic scale, improving local contrast while limiting the influence of outliers. In our experiments, this specific pre-processing proved essential to enable effective fusion under foggy or rainy conditions, where raw SWIR data would otherwise appear flat.
**HSV conversion.** The visible image is converted from the RGB to the HSV colour space to facilitate subsequent processing and yield better fusion results. This conversion isolates the hue (*H*), saturation (*S*), and value (*V*) components. The value component (*V*), which acts as the perceived visible luminance, is extracted and hereafter denoted as $I_{V}$ for the core fusion calculations. According to [33], colour spaces such as HSV or YCbCr give better visual results than RGB. Furthermore, HSV separates luminance from the chromatic channels, allowing colour saturation to be better preserved during the fusion process.

#### 2.1.2. Adaptive Weight Maps Calculation and Normalisation

The next step consists of building initial weight maps for each spectrum, $W_{\mathrm{VIS}}$ and $W_{\mathrm{SWIR}}$, using a combination of local metrics [24]. Although foundational, we detail their exact mathematical formulations below to clarify our specific implementation choices, including normalisation factors and numerical safeguards necessary for stable software deployment:

- **Local Contrast (Standard Deviation) *C*:** Assesses the high-frequency variability of intensities within a local neighbourhood $N_{1}$. To prevent numerical instabilities (e.g., negative variances due to floating-point precision limits), we apply a strict zero bound prior to the square root:
  (2) $$C\left(x,y\right)=\sqrt{\max\left(\mu_{I^{2}}\left(x,y\right)-{\left(\mu_{I}\left(x,y\right)\right)}^{2},\;0\right)}$$
  where $\mu_{I}$ denotes the local mean over $N_{1}$.
- **Local Entropy *J*:** Measures the statistical complexity and information content. For an 8-bit image domain, the maximum possible entropy is 8 bits. We explicitly normalise the standard Shannon entropy over a neighbourhood $N_{2}$ to bound the metric within [0,1]:
  (3) $$J\left(x,y\right)=-\frac{1}{8}\sum_{i=0}^{255}p_{i}\log_{2}\left(p_{i}\right)$$
  where $p_{i}$ is the probability of occurrence of intensity *i* within $N_{2}$.
- **Local Visibility *V*:** Estimates image clarity and local noise level by extracting the high-frequency residual from a first Gaussian blur ($G_{\sigma_{1}}$), and averaging its energy using a second Gaussian filter ($G_{\sigma_{2}}$):
  (4) $$V\left(x,y\right)=\sqrt{\left({\left[I-(I*G_{\sigma_{1}})\right]}^{2}*G_{\sigma_{2}}\right)\left(x,y\right)}$$
  where ∗ denotes the convolution operator.

In our implementation, to ensure spatial consistency while preserving sharp local boundaries, both $N_{1}$ and $N_{2}$ are defined as disk-shaped morphological neighbourhoods with a radius of 5 pixels. For the local visibility metric, both Gaussian filters ($G_{\sigma_{1}}$ and $G_{\sigma_{2}}$) utilise a 5×5 kernel size with a standard deviation of $\sigma_{1}=\sigma_{2}=2$.

Finally, these three resulting metric maps are equally weighted (α=1) and summed to form the raw, unscaled weight maps $W_{k}^{\mathrm{raw}}$ for each modality k∈{VIS,SWIR}:

(5) $$W_{k}^{\mathrm{raw}}\left(x,y\right)=C_{k}\left(x,y\right)+J_{k}\left(x,y\right)+V_{k}\left(x,y\right)$$

**Weighting factor and Normalisation.** In foggy or rainy conditions, the SWIR image contains most of the useful information, as illustrated in Figure 1. Consequently, an additional scaling should be applied to favour the SWIR image over the visible image. The weather-driven scaling factors $w_{\mathrm{VIS}}$ and $w_{\mathrm{SWIR}}$ are defined such that $w_{\mathrm{VIS}}=1-w_{\mathrm{SWIR}}$. The value of $w_{\mathrm{SWIR}}$ is determined through the control policy described in Section 2.2. Finally, the precise, normalised weight maps $W_{\mathrm{VIS}}$ and $W_{\mathrm{SWIR}}$ used for the subsequent multi-scale pyramid fusion are computed at each pixel (x,y) as follows:

(6) $$W_{k}\left(x,y\right)=\frac{w_{k}\cdot W_{k}^{\mathrm{raw}}\left(x,y\right)}{w_{\mathrm{VIS}}\cdot W_{\mathrm{VIS}}^{\mathrm{raw}}\left(x,y\right)+w_{\mathrm{SWIR}}\cdot W_{\mathrm{SWIR}}^{\mathrm{raw}}\left(x,y\right)}$$

This normalisation ensures a contextually balanced contribution between the two spectra based on the environmental context.

#### 2.1.3. Multi-Scale Pyramid Fusion

For the image fusion stage, the luminance images ($I_{V}$ representing the visible Value channel, and $I_{\mathrm{SWIR}}$ representing the pre-processed SWIR data) are decomposed into Laplacian pyramids ($\Lambda_{V}$ and $\Lambda_{\mathrm{SWIR}}$), while the weight maps are decomposed into Gaussian pyramids. The pyramids are composed of *L* levels. Gaussian pyramids are used to smooth images at different scales, while Laplacian pyramids capture details at each level of decomposition. The weight maps are used within the pyramid framework to adaptively guide the combination of information from the Visible and SWIR images, ensuring that the fusion process effectively integrates the most relevant data from each source. Following this, we compute the *l*-th level of the global pyramid $P^{l}$ using the following equation:

(7) $$P^{l}=W_{\mathrm{VIS}}^{l}⊙\Lambda_{V}^{l}+W_{\mathrm{SWIR}}^{l}⊙\Lambda_{\mathrm{SWIR}}^{l},$$

where $W_{\mathrm{VIS}}^{l}$ and $W_{\mathrm{SWIR}}^{l}$ are the *l*-th level of weight pyramids for the VIS and SWIR, and $\Lambda_{V}^{l}$ and $\Lambda_{\mathrm{SWIR}}^{l}$ are the *l*-th levels of the Laplacian pyramids of visible luminance and SWIR. Note that ⊙ is the element-wise multiplication.

A fused luminance pseudo-image *R* is recursively reconstructed by collapsing the global pyramid. The reconstruction proceeds from the coarsest level l=L to the finest l=1, and the luminance image $R^{l}$, at level *l*, is given by

(8) $$R^{l}=\mathrm{upsample}\;\left(R^{l+1}\right)+P^{l}\;;\;\mathrm{with}\;R^{L}=P^{L},$$

where $P^{l}$ is the current level of the global pyramid obtained from Equation (7) and the $\mathrm{upsample}\;\left(\cdot \right)$ is performed by creating an image of two-fold size with padding of two pixels convolved with a Gaussian filter.

The image is then reconstructed in HSV colour space, using hue (*H*) and saturation (*S*) from the visible image, and assigning the fused pseudo-luminance *R* to the value channel. The image is then converted back from HSV to RGB space to obtain a “traditional” colour image, $I_{F}$.

#### 2.1.4. Weather-Aware Post-Processing

**Figure 4:**
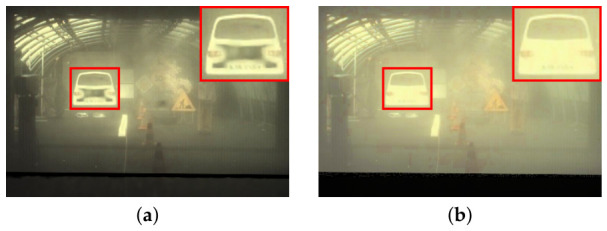
Visual comparison of pseudo-RGB images from our VISWIR method before (**a**) and after (**b**) post-processing. The latter shows enhanced visual quality, with improved local contrast, sharper details and reduced artefacts, while preserving the natural colour rendering of the fused image. The red boxes highlight specific regions where these structural and contrast enhancements are most prominent.

To restore a natural colour representation, a post-processing step is required because replacing the original visible luminance with the merged pseudo-luminance alters and saturates the colours (see Figure 4a). In [24], the image is corrected using a fixed coefficient, β=1.5. In our method, β is adjusted via offline parameter scheduling according to the weather-driven control policy defined in Section 2.2. Under low-intensity conditions, a higher β amplifies the contrast, enhancing the visibility of subtle details, whereas a lower β prevents over-saturation and maintains a balanced image under higher intensities. This strategy ensures that the fusion process adapts, via scheduled parameters, to the characteristics of the SWIR data, resulting in a more visually coherent and informative fused image.

An inverse tone mapping is thus applied to each colour channel *c* of the fused image $I_{F}$. This operation is performed as a global spatial overlay, not within the multi-scale decomposition. Non-linear transforms (e.g., the exponential factor β) on Laplacian pyramid coefficients (zero-mean, signed high-frequency values) distort structural relationships and create artefacts like ringing or halos. Accurate colour correction also requires the fully reconstructed spatial luminance.

Unlike the Pyramid Transform method [24], which did not weight each spectrum (i.e., $w_{\mathrm{SWIR}}=0.5$), our method assigns an adapted importance to the SWIR using the weighting factor defined in Section 2.1.2. It ensures that SWIR data are preserved throughout the fusion process. This prevents the inverse tone mapping from overriding the enhancements achieved during fusion, thereby maintaining consistency in colour correction without compromising the luminance fusion. Each channel *c* of the output image $I_{\mathrm{out}}$ is then computed as follows:

(9) $$I_{\mathrm{out}}^{c}={\left(I_{F}^{c}⊘\left(R+\epsilon \right)\right)}^{\circ \beta }⊙\left(w_{\mathrm{VIS}}\cdot I_{V}+w_{\mathrm{SWIR}}\cdot I_{\mathrm{SWIR}}\right),$$

where $I_{F}^{c}$ is the *c*-th channel of the fused image $I_{F}$, *R* is the reconstructed luminance image, and ϵ is a small constant (e.g., $\epsilon =10^{-12}$) added to prevent division by zero. The parameter β is the inverse tone mapping factor applied element-wise (denoted by the symbol ∘), $I_{V}$ and $I_{\mathrm{SWIR}}$ are the original visible and SWIR luminances, $w_{\mathrm{VIS}}$ and $w_{\mathrm{SWIR}}$ are the scalar weighting factors, and ⊘ denotes element-wise division.

Finally, a standard inverse gamma correction with parameter γ is applied to each channel *c* of $I_{\mathrm{out}}$ to adjust the global luminance response for standard display mapping:

(10) $$I_{\mathrm{display}}^{c}={\left(I_{\mathrm{out}}^{c}\right)}^{\circ \frac{1}{\gamma }}$$

This final adjustment ensures perceptually consistent brightness for human observation without altering the relative spatial contrast produced by the multi-scale fusion.

### 2.2. Weather-Driven Parameter Optimisation

To ensure robustness across varying environments, the fusion pipeline is governed by a “Weather-Driven Control Policy”. Unlike static tuning strategies, we establish a mapping between discrete weather categories (e.g., Clear, Rain, and Fog) and specific fusion hyperparameter sets, defined previously as $\Theta =\{w_{\mathrm{SWIR}},L,\beta ,\gamma \}$. To determine the optimal configuration for each set, we formulate an automated offline optimisation process utilising the Optuna framework (version 4.3.0) [34].

Instead of manually tuning the parameters, this process is modelled as a multi-objective optimisation problem. The goal is to maximise the perceptual quality of the fused image for specific weather categories. Using Optuna’s Tree-structured Parzen Estimator (TPE) sampler, the framework explores the continuous and discrete hyperparameter spaces to establish a Pareto front, balancing trade-offs between conflicting Non-Reference Image Quality Assessment (NR-IQA) metrics. Because the resulting Pareto front is generally non-convex, selecting a single optimal configuration requires a robust trade-off strategy. To achieve this, we identify the theoretical ideal point (or utopia point) for our metrics and select the solution on the Pareto front that minimises the Chebyshev distance ($L_{\infty }$ norm) to this ideal point. Unlike simple weighted sums or the Euclidean distance, which can fail to capture solutions in non-convex regions and allow one metric to be severely degraded if others compensate, the Chebyshev distance mathematically minimises the maximum deviation from the ideal point. This guarantees the best “worst-case” scenario, ensuring balanced perceptual quality across all conflicting metrics. This approach guarantees an optimal balance that maximises the total information content (here, entropy [35]) while simultaneously minimising perceptual distortions (evaluated via NIQE [36], PIQE [37], and BRISQUE [38], as detailed in Section 3.3).

This discrete mapping is extensible to a finer granularity (e.g., distinguishing light versus dense fog) without architectural changes. It ensures that the algorithm adapts its behaviour to the constraints of each environment: for instance, the $\Theta_{\mathrm{Fog}}$ set is automatically configured by Optuna to prioritise the penetrative sensor (higher $w_{\mathrm{SWIR}}$) and adjust tone mapping β to maintain local contrast, whereas $\Theta_{\mathrm{Clear}}$ favours a more natural colour rendering.

From an embedded perspective, this works as a Look-Up Table (LUT) scheme where a weather-sensing module identifies the environmental state and retrieves the corresponding pre-optimised parameter set, enabling context-aware parameter switching during deployment without manual user intervention. However, as documented in the video processing literature addressing frame-by-frame temporal consistency [39], independent processing often leads to visual instability in dynamic scenes. To avoid visual flickering during weather transitions in a real-time embedded system, a direct temporal interpolation of the Θ parameters is impractical, primarily because the pyramid depth (*L*) is a strictly discrete structural variable. Instead, a robust deployment strategy would require applying temporal hysteresis to the weather classifier’s decisions to prevent state oscillation, coupled with a temporal cross-fading (alpha blending) of the fused image outputs over a short sliding window when transitioning between two predefined parameter sets.

## 3. Experimental Setup and Evaluation

### 3.1. Dataset and Experimental Setup

**Figure 5:**
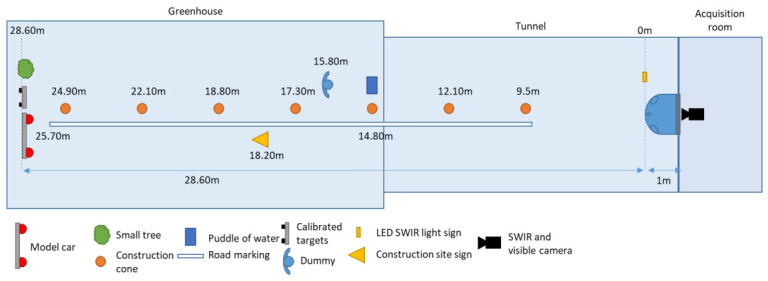
Illustration of the experimental setup used at the Cerema PAVIN platform. Adapted from [9].

To validate the proposed fusion method, a series of experiments were conducted under various environmental conditions at the Cerema’s PAVIN “Fog & Rain” platform [8], which enables the controlled generation of fog and rain at different intensity levels, both during the day and at night. This setup, illustrated in Figure 5, allowed us to acquire multi-modal datasets in a reproducible and consistent environment with synchronised meteorological metrics.

All acquisitions were performed in a static scene configuration to ensure consistent framing and minimise motion-related variability. While the physical scene composition remains static to guarantee accurate pixel-level alignment between modalities, the dynamic nature of the dataset is driven entirely by the continuously evolving weather conditions. All image pairs are registered: temporal registration was performed using image timestamps, and visual registration was achieved using LightGlue [40].

**Sensors.** Two cameras were used: a visible camera (Teledyne Dalsa, Waterloo, ON, Canada; Genie Nano C1630; 1632 × 1248 resolution) and a SWIR camera [10] (SWIR Vision Systems, Morrisville, NC, USA; Acuros^®^ CQD^®^ 1280; 1280 × 1024 resolution). Notably, this study leverages Colloidal Quantum Dot (CQD) technology for the SWIR sensor, offering a high-resolution alternative to the traditional InGaAs sensors predominantly used in the existing literature. While this fine spatial resolution is essential for effective deep multi-scale decomposition, an in-depth radiometric comparison between CQD and InGaAs remains beyond the algorithmic scope of this work. A comprehensive hardware evaluation detailing the signal-to-noise ratio (SNR) and dynamic range of these technologies can be found in the manufacturer’s comparative analysis [41]. Furthermore, the concrete benefits of this specific CQD sensor for downstream perception tasks (e.g., object detection and segmentation) under adverse weather conditions have been previously validated in recent experimental studies [4].
**Meteorological Conditions.** Our dataset presented in [4] is composed of a diverse set of images acquired in clear weather (15 images at 1 frame per second), foggy conditions (86 images at 1 frame every 5 s), and rainy scenarios (125 images at 1 frame every 5 s). Weather conditions ranged from clear visibility to dense fog (visibility from 10 m to 400 m) and rain with intensities from 20 to 170 mm/h. A distinctive feature of this dataset is the inclusion of precise meteorological metrics (e.g., exact visibility range and rainfall rate) for every individual frame. This provides a synchronised environmental ground truth that is rarely available in standard public datasets.

### 3.2. Baselines and Implementation Details

We evaluated our method against four fusion baselines: Top-Hat [23], TarDAL [18], V-SWIR-IF [26], and the foundational work of [24], hereafter referred to as “Pyramid Transform”. We utilised the official source codes for TarDAL and Pyramid Transform. We have re-implemented, to the best of our capacity, V-SWIR-IF and Top-Hat, as no public implementation was available.

To ensure a strictly fair comparison, all methods were evaluated using identical input data pipelines. Furthermore, to prevent any evaluation bias, the baseline models were deployed “off-the-shelf” without any dataset-specific fine-tuning, strictly adhering to the default parameter configurations recommended by their original authors.

It is worth noting that while TarDAL was originally designed for visible and Long-Wave Infrared (LWIR) fusion, a domain governed by thermal emissivity, it remains a robust, state-of-the-art infrared baseline. Its inclusion deliberately highlights the physical differences and methodological challenges encountered when applying emissive-tailored architectures to the reflective SWIR spectrum.

Other approaches, such as [27] or [28], could not be evaluated due to the lack of public access to implementation details.

### 3.3. Evaluation Metrics

Evaluating image fusion in adverse weather conditions is inherently challenging due to the physical impossibility of capturing flawless, ground-truth reference images in such dynamic environments. For this reason, standard colour distortion metrics (such as CIEDE2000 or ΔE) were deliberately excluded.

Instead, we rely on four well-established No-Reference Image Quality Assessment (NR-IQA) metrics. This choice allows the evaluation protocol to be applied seamlessly to other datasets and real-world scenarios where pristine references are unavailable. To ensure a comprehensive assessment, each metric captures a distinct aspect of perceptual quality. Crucially, several of these leverage Natural Scene Statistics (NSS) to explicitly quantify perceptual “naturalness”. By measuring how much an image’s spatial statistical distributions deviate from mathematical models of distortion-free scenes, they provide an objective estimation of both structural and chromatic anomalies:

- **Normalised Entropy (NE) (↑)** [35]: This metric estimates the total information content and signal complexity within an image. In the context of multi-spectral fusion, a higher NE score indicates that a greater amount of structural detail and texture from both the visible and SWIR sources has been successfully preserved in the final combined image.
- **BRISQUE (↓)** [38]: The Blind/Referenceless Image Spatial Quality Evaluator assesses the overall naturalness of an image. It relies on a Support Vector Regression (SVR) model trained on Natural Scene Statistics (NSS) to quantify losses in naturalness. A lower score signifies that the fused image exhibits fewer unnatural artefacts and more closely resembles a clear, undistorted scene.
- **NIQE (↓)** [36]: The Natural Image Quality Evaluator also leverages NSS but, unlike BRISQUE, operates completely blindly without requiring prior training on distorted images or human subjective scores. It measures the statistical distance between the evaluated image and a corpus of high-fidelity natural images. A lower NIQE value indicates higher perceptual quality and fewer spatial distortions.
- **PIQE (↓)** [37]: The Perception-based Image Quality Evaluator is an unsupervised metric that evaluates locally perceptible distortions by analysing local block variances. It is primarily designed to penalise blur and blockiness (e.g., compression artefacts). A lower PIQE score reflects an image with high apparent sharpness and distinct edges, though it does not explicitly differentiate between true structural texture and high-frequency sensor noise.

Together, these metrics provide a complementary view of perceptual quality that is directly relevant to autonomous vehicle perception: higher NE and lower BRISQUE, NIQE, and PIQE values generally correspond to clearer, more informative imagery, which can benefit downstream tasks.

## 4. Results

This section presents the evaluation of the proposed VISWIR framework. First, the optimised weather-driven parameters obtained through the Optuna framework are detailed, illustrating the contextual adaptability of the method. Subsequently, to assess the visual impact of the approach, a qualitative comparison against baseline fusion methods is provided under adverse weather conditions. Finally, a quantitative evaluation based on the previously introduced non-reference metrics is conducted across various meteorological categories.

### 4.1. Optimised Weather-Driven Parameters

The automated optimisation process via Optuna yielded specific hyperparameter configurations for three distinct weather categories: Clear, Rain, and Fog. To ensure robust generalisation and prevent overfitting, the image pairs within each category were stratified into an 80% training split, used to drive the objective function evaluation, and a 20% validation split. Table 1 summarises the optimal values obtained for the weighting factor ($w_{\mathrm{SWIR}}$), pyramid depth (*L*), inverse tone mapping factor (β), and gamma correction (γ) across these conditions.

It is worth noting that this systematic exploration of the parameter space also acts as an implicit ablation study. By evaluating the Pareto front across varying conditions, it demonstrates that static, conventional settings are often suboptimal, thereby justifying our context-aware design choices without the need for additional manual ablations.

These optimised values reflect the distinct physical properties of each spectrum under atmospheric disturbances. The global factor $w_{\mathrm{SWIR}}$ remains high across all contexts (ranging from 0.85 to 0.90), showing that the optimisation framework prioritises the structural robustness of the SWIR sensor. The inverse tone mapping exponent β scales monotonically with weather severity, rising from 1.07 in clear conditions to 1.59 in rain and peaking at 2.31 in dense fog. This adjustment stretches the dynamic range to recover low-contrast structural details heavily compressed by atmospheric scattering. The optimal pyramid depth *L* decreases from 5 (Clear) to 1 (Fog). A deep multi-scale decomposition (L=5) smoothly blends rich natural textures in clear weather. Interestingly, the drop to a shallow architecture (L=1) under severe fog was not a manual heuristic but the optimal parameter automatically discovered by the framework. This aligns perfectly with optical physics: in dense fog, Mie scattering acts as a severe low-pass filter on the visible spectrum, heavily attenuating high frequencies and blurring structural edges. Because a deep pyramid propagates low-frequency information across spatial scales, keeping *L* high would risk contaminating the sharp, penetrative structures captured by the SWIR sensor with the atmospheric blur from the visible band. By dropping to L=1, the framework physically isolates the SWIR structure and restricts fusion to immediate, high-frequency pixel-level gradients, effectively preventing blur contamination.

In our experimental setup, the meteorological condition for each sequence is known a priori, allowing us to statically assign the corresponding optimised parameter set. However, for real-world autonomous navigation, this context must be inferred automatically during deployment. To achieve this, the proposed framework can be coupled with real-time weather attribute detection modules. Recent advancements, such as the heuristic and style-based neural architectures proposed by Ouattara et al. [42], demonstrate efficient weather classification. Integrating such a sensing module could theoretically enable our system to transition between hyperparameter sets (e.g., from $\Theta_{\mathrm{Clear}}$ to $\Theta_{\mathrm{Fog}}$) in real time as environmental conditions evolve, offering a finer granularity of adaptation without requiring manual intervention.

### 4.2. Qualitative Evaluations

**Figure 6:**
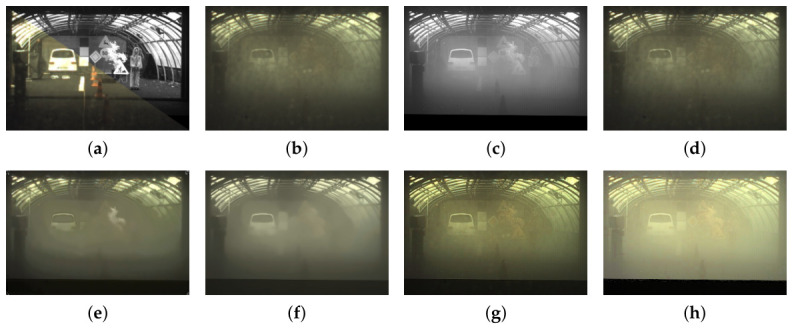
Visual comparison of image fusion methods applied to the same scene under average fog conditions (28 m visibility): (**a**) Clear (VIS/SWIR) reference; (**b**) Visible image; (**c**) SWIR image; (**d**) Top-Hat [23]; (**e**) TarDAL [18]; (**f**) V-SWIR-IF [26]; (**g**) Pyramid [24]; (**h**) VISWIR (**Ours**).

Our qualitative analysis focuses on the ability of each method to preserve fine details, to maintain natural colour rendering, and to enhance scene visibility when the visible spectrum alone is degraded. Visual comparisons, shown in Figure 6, illustrate the differences in detail preservation and contrast under a harsh weather scenario. To establish a baseline, the first image of the figure displays the scene under clear weather conditions, followed by the degraded VIS and SWIR inputs under average fog.

Observing the fusion baselines, Top-Hat preserves natural colours and enhances some structures, but underexploits the complementary information brought by SWIR, yielding limited gains in contrast and fine-detail visibility under adverse conditions. TarDAL reveals several scene elements but does not fully preserve colour information, resulting in some objects being clearly visible while others remain difficult to discern. V-SWIR-IF produces a flatter overall colour rendering, and some details, such as the pedestrian on the right side of the scene, remain challenging to identify. Finally, the Pyramid Transform method produces a visually sharp image with high contrast, but this sharpness is largely due to the preservation of high-frequency sensor noise. It results in a granular texture that gives a “gritty” appearance rather than recovering true structural details, which is usually required by higher-level perception tasks, such as object detection and scene understanding.

In contrast, within the scope of the evaluated adverse weather conditions, VISWIR demonstrates a consistent improvement in perceptual quality. Unlike the baseline Pyramid Transform, visual inspections confirm that it effectively suppresses sensor noise while improving local contrast, producing well-balanced, realistic colours.

**Figure 7:**
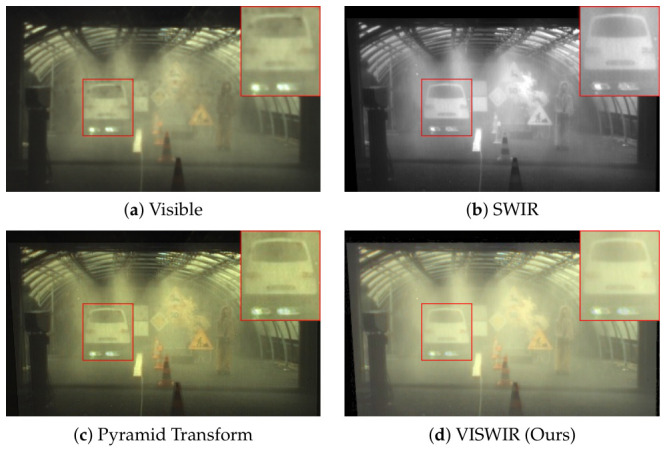
Qualitative comparison under average rain conditions (87 mm/h). The visible (**a**) and SWIR (**b**) inputs suffer from atmospheric scattering and low contrast, respectively. The Pyramid Transform baseline (**c**) introduces pervasive granular noise and localised dark artefacts. Conversely, the proposed VISWIR framework (**d**) suppresses sensor noise and recovers structural details, yielding a smoother and more natural rendering. The red boxes denote the vehicle’s close-up regions, explicitly highlighting the severe noise of the baseline compared to the smooth VISWIR output.

To explicitly highlight these differences against the closest baseline, Figure 7 presents a targeted comparison under moderate rain conditions (87 mm/h). As evidenced by the close-up on the vehicle, the Pyramid Transform output exhibits a harsh, granular texture accompanied by small localised dark artefacts. Consequently, fine semantic details, such as the deer warning sign, are degraded by this noise, and the foreground shadows remain difficult to interpret. Conversely, VISWIR maintains a smoother structural rendering devoid of these artificial artefacts. It successfully recovers visibility in the underexposed foreground regions, allowing scene elements and semantic cues to remain more distinguishable. Rather than artificially over-enhancing the contrast, which often amplifies noise, VISWIR yields a slightly diffuse rendering. This physically reflects the natural atmospheric scattering (i.e., the rain curtain) captured by the SWIR sensor, resulting in a cleaner and more realistic representation of the environment.

Overall, the SWIR contribution enables the generation of pseudo-images that preserve both fine details and colour fidelity, making scene elements more clearly distinguishable without introducing artificial noise. This visual stability was consistently observed across all specific weather scenarios present in our dataset, ranging from light rain to dense fog.

### 4.3. Quantitative Evaluations

To corroborate the visual assessments, a comprehensive quantitative analysis was conducted. Table 2, Table 3, Table 4 and Table 5 summarise the impact of varying weather conditions on image quality across the four evaluated NR-IQA metrics.

**Clear Weather.** Our method achieves the best scores for both NE↑ (0.638) and NIQE↓ (8.74) and ranks second for BRISQUE↓ (8.23) and PIQE↓ (36.75). This is a critical result demonstrating that VISWIR does not introduce artificial degradation when the input images are already of high quality. It acts as a non-destructive enhancement layer, unlike certain dehazing methods that inject false contrast or saturation artefacts in the absence of atmospheric scattering.
**Rain Scenarios.** VISWIR proves to be the most consistent method across varying rain intensities. It improves BRISQUE↓ over the visible spectrum alone in both light and average rain (e.g., from 28.60 to 16.39 in average rain) and achieves the best NIQE↓ scores overall. Comparatively, Top-Hat often yields lower PIQE↓ scores but fails to significantly improve BRISQUE↓, while TarDAL exhibits unstable behaviour across different rain severities.
**Light Fog (102 m visibility).** VISWIR remains highly competitive, ranking second in NIQE↓ (8.75) and achieving a BRISQUE↓ score (8.29) comparable to the best-performing methods. Notably, even under these mild conditions, Pyramid Transform begins to show limitations in naturalness (BRISQUE 13.68), suggesting that its static fusion logic struggles to balance spectral contributions as effectively as our weather-optimised weighting.
**Average Fog (28 m visibility).** As conditions deteriorate and visibility drops, VISWIR achieves a balanced performance, securing the best NIQE↓ (9.04) and competitive NE↑ results. Although Pyramid Transform shows strong entropy scores here, its significantly higher BRISQUE↓ (38.90 vs. 12.20 for VISWIR) suggests that the measured “information” contains substantial perceptual distortions, which are heavily penalised by natural scene statistics metrics.
**Heavy Fog (15 m visibility).** The advantage of the proposed approach is most evident in this extreme scenario. Pyramid Transform suffers a severe degradation with a BRISQUE↓ score of 72.23 (worse than the visible image at 47.17), indicating a failure to handle low-contrast SWIR data. In contrast, VISWIR maintains a score of 17.02, closely tracking the best score obtained by the SWIR sensor alone (10.91). This confirms that without specific pre-processing and adaptive weighting, standard pyramid fusion cannot cope with extreme signal attenuation. While Pyramid Transform achieves a slightly higher NE↑, this metric does not discriminate between true structural details and the high-frequency sensor noise that the baseline preserves.
**Local Distortion (PIQE) Interpretation.** As observed in Table 5, Pyramid Transform frequently achieves the lowest PIQE scores. However, as previously illustrated in the qualitative analysis (Figure 6), this baseline produces a significant high-frequency grain. Because PIQE is a no-reference metric primarily designed to penalise blur and blockiness, it often misinterprets this pervasive sensor noise as “texture” or “sharpness”, resulting in an optimistically low distortion score. This apparent sharpness comes at the cost of overall perceptual naturalness (reflected by its poor BRISQUE performance). Conversely, VISWIR applies adaptive contrast clipping to suppress this noise, resulting in a smoother, cleaner image. While this reduction in high-frequency grain leads to a comparatively higher PIQE score, it represents a truthful restoration of the scene’s structural content without artificial noise injection.
**Summary.** Across the full spectrum of evaluated weather scenarios, VISWIR achieves competitive overall stability instead of delivering optimal performance across all metrics and scenarios. As evidenced by the PIQE and BRISQUE scores, where certain baselines occasionally peak by favouring high-frequency noise or specific contrast features, VISWIR prioritises a balanced trade-off. Unlike baseline methods that excel in one specific metric or weather condition but significantly underperform in another, VISWIR avoids extreme variations. This stability directly validates the effectiveness of the weather-aware parameter scheduling, ensuring that the fusion framework provides a reliable and perceptually coherent perception stream for autonomous systems, regardless of environmental severity.

## 5. Discussions

### 5.1. Performance Analysis and Methodological Limitations

These quantitative results confirm the effectiveness of our VISWIR method and highlight the potential of VIS–SWIR image fusion to improve perception in harsh environmental conditions.

**Offline Optimisation and Extreme Degradation.** While our current “weather-driven parameter scheduling” significantly enhances robustness compared to fixed baselines, it is important to clarify that the parameter optimisation process (via Optuna) is executed entirely offline to generate discrete sets of hyperparameters. Consequently, during inference, the system selects ready-made parameters from a categorical look-up table rather than computing them in real time, which slightly limits the strict definition of being “dynamically adaptive”. Furthermore, limitations are still observed under extreme degradation (e.g., dense fog with visibility under 15 m), where the signal itself is severely attenuated. Future work could investigate continuous, real-time parameter optimisation to handle these edge cases more effectively than the current discrete approach.
**Metrics.** It should also be noted that the metrics used in this study are designed for, and in some cases trained on, visible images and are not specific to SWIR. This can inherently favour visible images in the evaluation process and, in some cases, lead to counter-intuitive results. For example, in the case of PIQE in average fog, the smoothing effect of fog can reduce local variance and yield lower (better) scores for visible images, even when SWIR reveals more scene details to a human observer. Similarly, BRISQUE relies on Natural Scene Statistics (NSS) that multispectral fusion images (VIS–IR or VIS–SWIR) may deviate from, especially when high-frequency noise is preserved, leading to an overestimation of perceived degradation. To reduce metric sensitivity to high-frequency noise, light denoising was applied to the luminance channel of TarDAL and V-SWIR-IF outputs before evaluation, without affecting colour information. Our method and Top-Hat did not require this adjustment. Therefore, future research should develop customised metrics that better reflect the characteristics of SWIR imagery and provide a more balanced assessment of fusion quality across spectral domains.
**Computational Efficiency.** Execution times were measured on a standard CPU architecture (Intel i7) using 1620×925 images. The proposed CPU implementation averages 0.70 s per pair, being slightly faster than the Pyramid Transform method (∼0.77 s) and being significantly faster than Top-Hat and V-SWIR-IF (which both require ∼2.5 s). Notably, despite running entirely on a CPU, it remains competitive with the GPU-accelerated TarDAL (<0.5 s). This confirms the algorithmic efficiency of VISWIR on standard hardware, establishing it as a strong, lightweight algorithmic baseline for future embedded perception systems rather than a fully deployed real-time solution.

However, while these static evaluations and runtime metrics establish a strong foundation, they inherently limit the assessment of the algorithm’s robustness in real-world driving conditions, motivating our subsequent exploration on dynamic datasets.

### 5.2. Dynamic Scenario Assessment

The quantitative experiments were primarily performed on static scenes with controlled dynamic weather conditions. However, VISWIR is fully compatible with dynamic environments containing moving objects, such as pedestrians or vehicles. Because our method performs a purely spatial, pixel-level fusion on a frame-by-frame basis without any temporal accumulation, it inherently avoids the algorithmic motion blur or ghosting artefacts often introduced by temporal filtering methods. Consequently, any motion blur or ghosting present in the fused output would strictly stem from the hardware’s sensor exposure time or slight imperfections in the spatial registration process, provided that accurate hardware synchronisation between the sensors is maintained.

To address the utility of the proposed method in dynamic, real-world driving scenarios, preliminary tests were performed on a subset of the RASMD dataset [14], named RASMD-IP and introduced in [43]. The environment-specific parameters, $\Theta_{\mathrm{RASMD}}$, were obtained using the exact same optimisation methodology previously described in Section 2.2. To prevent any data leakage, the optimisation was executed strictly on the RASMD-IP training set (1524 images), focusing on a high-dynamic-range subset, while the quantitative metrics were evaluated exclusively on the separate, unseen test set (385 images).

The automated optimisation for this specific high-dynamic-range context yielded the following hyperparameter set: $w_{\mathrm{SWIR}}=1.0$, L=4, β=4.62, and γ=1.88. These values strongly reflect the physical constraints of the scene. The algorithm assigns maximum structural weight to the SWIR spectrum ($w_{\mathrm{SWIR}}=1.0$), effectively discarding the underexposed visible luminance, while applying an aggressive inverse tone mapping (β=4.62) to stretch the compressed dynamic range within deep shadows. The visible spectrum, in this context, is primarily leveraged to colourise the SWIR structural backbone.

**Figure 8:**
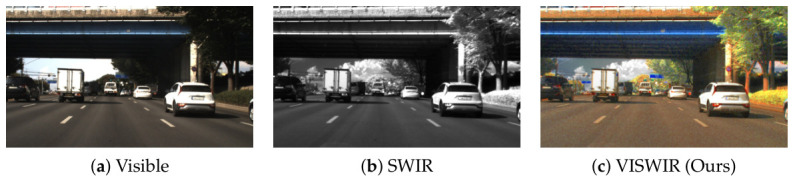
Qualitative evaluation on the RASMD dataset in a high-dynamic-range scenario. The visible image (**a**) suffers from underexposure under the bridge, while the SWIR image (**b**) highlights the high reflectance of the vegetation. The IQA-optimised VISWIR fusion (**c**) integrates both, recovering details in deep shadows without saturating the bright areas.

This behaviour is clearly illustrated in Figure 8. In a scenario featuring deep shadows under a bridge, the visible sensor loses structural information in the dark areas, while the SWIR sensor captures the bright reflectance of the surrounding vegetation. The IQA-optimised VISWIR framework fuses these modalities, recovering the hidden details under the bridge while maintaining the high-contrast vegetation and natural colour rendering.

To substantiate these qualitative observations, Table 6 presents NR-IQA metrics evaluated on a high-dynamic-range subset of the RASMD-IP test set. The fusion improves the overall information content (highest entropy) and reduces local distortions (lowest PIQE) compared to the standalone visible and SWIR images.

These preliminary findings on the RASMD dataset corroborate the qualitative observations previously done. They demonstrate that the VISWIR framework extends its context-adaptive fusion capabilities to dynamic, high-contrast environments, providing a robust perceptual foundation for autonomous navigation systems.

## 6. Conclusions

This paper addresses the challenge of robust perception for autonomous vehicles in adverse weather, where conventional visible cameras suffer from severe degradations. To overcome this limitation, we introduce VISWIR, a VIS–SWIR image fusion method designed to enhance scene visibility, preserve fine details, and maintain natural colour rendering across diverse conditions. By adapting a weight-map-guided pyramidal fusion framework and optimising its parameters to suit weather conditions using our Weather-Driven Parameters Control module, VISWIR expands the ODD of vision-based perception systems.

Experimental validation across diverse meteorological scenarios demonstrates that VISWIR improves perceptual quality compared to individual modalities and alternative fusion methods. Furthermore, run-time analysis confirms the algorithmic efficiency of the approach, supporting its scalability for robotic constraints. Moreover, because the core local metrics (such as variance and entropy) operate on normalised statistical intensity distributions rather than absolute radiometric values, the framework is mathematically agnostic to specific wavelengths.

Consequently, it possesses broad generalisability and could be seamlessly extended to other sensor combinations, such as VIS-NIR or VIS-LWIR, provided the discrete parameter set (Θ) is re-optimised for the physical properties of the respective spectra. While NR-IQA optimisation greatly benefits human interpretability, we observed a dichotomy: statistically altering images to maximise visual comfort does not consistently align with the feature extraction mechanisms of standard, pre-trained neural networks.

Future work will focus on further optimising VISWIR’s computational footprint for embedded deployment and achieving full system autonomy by integrating a closed-loop, real-time weather classifier [42] to automatically drive parameter switching. To bridge the gap between human and machine perception, we plan to investigate task-driven parameter scheduling. By evaluating higher-level tasks such as object detection and semantic segmentation, we aim to develop alternative fusion configurations explicitly tailored to the structural requirements of autonomous navigation algorithms in diverse operational scenarios.

## Figures and Tables

**Table 1:** Optimised hyperparameter sets for different weather conditions obtained for our Weather-Driven Parameters Control module. The predefined search space bounds for each parameter are indicated in the second row. During optimisation, the continuous variables ($w_{\mathrm{SWIR}}$, β, γ) were sampled with a step size of 0.01, while the discrete pyramid depth (*L*) was sampled with a step of 1.

Weather Condition	$w_{\mathrm{SWIR}}$	*L*	β	γ
Search Space	[0.0, 1.0]	{1, …, 6}	[1.0, 5.0]	[0.01, 4.0]
Clear	0.89	5	1.07	2.82
Rain	0.85	3	1.59	1.77
Fog	0.90	1	2.31	2.13

**Table 2:** Normalised Entropy (NE) results for all tested methods. The upward arrow (↑) indicates that higher values represent better performance (greater information content). Values in **bold** denote the best results, and underlined values indicate the second best.

Weather Conditions (Visibility (m) or Rainfall Rate (mm/h))	NE ↑						
	VIS	SWIR	TarDAL	Top-Hat	Pyramid Transform	V-SWIR-IF	VISWIR (Ours)
Clear weather (30 km; 0 mm/h)	0.610	0.602	0.606	0.607	0.611	0.619	**0.638**
Light rain (51 mm/h)	0.657	0.632	0.648	0.652	0.663	**0.664**	0.650
Average rain (87 mm/h)	0.629	0.640	**0.647**	0.628	0.646	0.639	0.639
Heavy rain (174 mm/h)	0.633	0.616	0.636	0.624	**0.644**	0.637	0.640
Light fog (102 m)	0.646	0.616	**0.657**	0.646	0.652	0.650	0.637
Average fog (28 m)	0.613	0.612	0.605	0.599	**0.631**	0.620	0.620
Heavy fog (15 m)	0.515	**0.603**	0.597	0.491	0.589	0.506	0.588
All weather (mean ± std)	0.615 ± 0.05	0.617 ± 0.01	0.628 ± 0.02	0.607 ± 0.05	**0.634 ± 0.03**	0.619 ± 0.05	0.630 ± 0.02

**Table 3:** Blind/Referenceless Image Spatial Quality Evaluator (BRISQUE) results for all tested methods. The downward arrow (↓) indicates that lower values represent better perceptual quality. Values in **bold** denote the best results, and underlined values indicate the second best.

Weather Conditions (Visibility (m) or Rainfall Rate (mm/h))	BRISQUE ↓						
	VIS	SWIR	TarDAL	Top-Hat	Pyramid Trans.	V-SWIR-IF	VISWIR (Ours)
Clear weather (30 km; 0 mm/h)	10.30	9.77	15.64	**6.75**	8.50	12.13	8.23
Light rain (51 mm/h)	12.55	**8.27**	9.04	10.24	9.18	8.34	9.93
Average rain (87 mm/h)	28.60	**11.78**	33.09	20.36	34.37	44.31	16.39
Heavy rain (174 mm/h)	9.66	**9.12**	15.03	10.82	21.27	34.93	10.31
Light fog (102 m)	12.53	10.44	**8.14**	8.16	13.68	23.20	8.29
Average fog (28 m)	23.06	**9.84**	38.44	19.39	38.90	55.98	12.20
Heavy fog (15 m)	47.17	**10.91**	51.35	43.27	72.23	91.92	17.02
All weather (mean ± std)	20.55 ± 13.73	**10.02 ± 1.16**	24.39 ± 16.65	17.00 ± 12.74	28.30 ± 22.73	38.69 ± 29.00	11.77 ± 3.63

**Table 4:** Naturalness Image Quality Evaluator (NIQE) results for all tested methods. The downward arrow (↓) indicates that lower values represent better perceptual quality. Values in **bold** denote the best results, and underlined values indicate the second best.

Weather Conditions (Visibility (m) or Rainfall Rate (mm/h))	NIQE ↓						
	VIS	SWIR	TarDAL	Top-Hat	Pyramid Transform	V-SWIR-IF	VISWIR (Ours)
Clear weather (30 km; 0 mm/h)	10.32	9.82	10.20	10.24	10.19	10.15	**8.74**
Light rain (51 mm/h)	9.76	9.72	9.92	9.82	10.04	10.53	**9.53**
Average rain (87 mm/h)	11.30	11.87	12.03	12.27	11.52	12.37	**11.10**
Heavy rain (174 mm/h)	9.78	9.86	11.50	11.68	10.75	11.53	**9.29**
Light fog (102 m)	9.40	**8.60**	10.65	9.48	9.33	9.40	8.75
Average fog (28 m)	10.94	9.28	11.81	11.54	9.93	10.94	**9.04**
Heavy fog (15 m)	12.56	**10.58**	12.41	12.76	11.07	12.79	11.88
All weather (mean ± std)	10.58 ± 1.11	9.96 ± 1.04	11.22 ± 0.96	11.11 ± 1.27	10.40 ± 0.75	11.10 ± 1.21	**9.76 ± 1.23**

**Table 5:** Perception-based Image Quality Evaluator (PIQE) results for all tested methods. The downward arrow (↓) indicates that lower values represent fewer locally perceptible distortions. Values in **bold** denote the best results, and underlined values indicate the second best.

Weather Conditions (Visibility (m) or Rainfall Rate (mm/h))	PIQE ↓						
	VIS	SWIR	TarDAL	Top-Hat	Pyramid Transform	V-SWIR-IF	VISWIR (Ours)
Clear weather (30 km; 0 mm/h)	41.41	43.02	60.29	50.51	**32.37**	58.50	36.75
Light rain (51 mm/h)	48.44	39.55	56.05	46.26	**36.00**	61.85	42.16
Average rain (87 mm/h)	41.77	36.00	65.73	49.02	**27.90**	70.22	37.20
Heavy rain (174 mm/h)	49.46	36.02	56.87	43.11	**28.98**	68.14	39.21
Light fog (102 m)	45.07	46.22	57.16	41.85	**32.83**	60.46	41.96
Average fog (28 m)	32.38	36.81	57.10	27.28	**24.56**	51.76	36.27
Heavy fog (15 m)	36.25	19.00	65.13	44.28	**17.23**	61.83	18.76
All weather (mean ± std)	42.11 ± 6.22	36.66 ± 8.69	59.76 ± 4.10	43.19 ± 7.67	**28.55 ± 6.23**	61.82 ± 6.12	36.04 ± 7.99

**Table 6:** NR-IQA evaluation on the high-dynamic-range subset (test set). The upward arrow (↑) indicates that higher values represent better performance, while the downward arrow (↓) indicates that lower values are better. Best results are in bold.

Modality	Entropy (↑)	NIQE (↓)	PIQE (↓)	BRISQUE (↓)
Visible Image	0.61	8.98	38.59	45.21
SWIR Image	0.61	**8.68**	51.61	48.35
VISWIR (Ours)	**0.64**	8.93	**35.59**	**38.65**

## Data Availability

The source code for the proposed VISWIR method is publicly available on GitHub (version 1.0) at https://github.com/comsee-research/VISWIR (accessed on 22 June 2026).The PAVIN datasets generated and analysed during the current study are not publicly available but are available from the corresponding author on reasonable request. The RASMD dataset [14] used for dynamic evaluation is publicly available at https://huggingface.co/datasets/STL-Yonsei/RASMD (accessed on 22 June 2026).
